# Positive impact of early-probiotic administration on performance parameters, intestinal health and microbiota populations in broiler chickens

**DOI:** 10.1016/j.psj.2024.104401

**Published:** 2024-10-10

**Authors:** M. Hussain, O. Aizpurua, A. Pérez de Rozas, N. París, M. Guivernau, A. Jofré, N. Tous, Z.W. Ng'ang'a, A. Alberdi, E. Rodríguez-Gallego, M.H. Kogut, J. Tarradas

**Affiliations:** ⁎ IRTA, Animal Nutrition, Mas Bové, 43120 Constantí, Catalonia, Spain; † Center for Evolutionary Hologenomics, Globe Institute, University of Copenhagen, Copenhagen, Denmark; ‡ IRTA, Animal Health, Centre de Recerca en Sanitat Animal (CReSA), Campus de la Universitat Autònoma de Barcelona (UAB), 08193 Bellaterra, Catalonia, Spain; § IRTA, Sustainability in Biosystems, Torre Marimón, 08140 Caldes de Montbui, Catalonia, Spain; # IRTA, Food Safety and Functionality, Finca Camps i Armet, 17121 Monells, Catalonia, Spain; ǁ MoBioFood Research Group, Department of Biochemistry and Biotechnology, Universitat Rovira i Virgili, c/Marcel·lí Domingo n°1, 43007 Tarragona, Spain; ¶ Southern Plains Agricultural Research Center, USDA-ARS, College Station, TX, USA

**Keywords:** probiotic, intestinal health, poultry, gut inflammation, microbiota

## Abstract

Minimizing the utilization of antibiotics in animal production is crucial to prevent the emergence of antimicrobial resistances. Thus, research on alternatives is needed to maintain productivity, sustainability, and animal health. To gain a comprehensive understanding of probiotics’ modes of action on performance, intestinal microbiota, and gut health in poultry, 3 probiotic strains (*Enterococcus faecalis* CV1028 [***EntF***]*, Bacteroides fragilis* GP1764 [***BacF***], and *Ligilactobacillus salivarius* CTC2197 [***LacS***]) were tested in 2 in vivo trials. Trial 1 comprised of a negative control group fed basal diet (**BD**) and 3 treatment groups that received BD with *EntF, BacF* and *LacS*. Trial 2 included a negative control group, a positive control group with Zinc-Bacitracin as antibiotic growth promoter **(AGP)**, and 2 groups treated with a blend of probiotics (*EntF+BacF+LacS*) during 0 to 10 or 0 to 35 d, respectively. Wheat-soybean-rye based diets without exogenous enzymes were used as a challenge model to induce intestinal mild- or moderate-inflammatory process in the gut.

In Trial 1, individually administered probiotics improved FCR at 8 d compared to Control, but these positive effects were lost in the following growing periods probably due to the high grade of challenging diet and a too low dose of probiotics. In Trial 2**,** both Probiotic treatments, administered only 10 or 35 d, significantly improved FCR to the same extent as of the Antibiotic group at the end of the trial. Although the performance between antibiotic and probiotic mixture showed similar values, microbiota analysis revealed different microbial composition at 7 d, but not at 21 d. This suggests that modes of action of the AGP and the tested probiotic blend differ on their effects on microbiome, and that the changes observed during the first days’ posthatch are relevant on performance at the end of the study. Therefore, the probiotics administration only during the first 10 d posthatch was proven sufficient to induce similar performance improvements to those observed in birds fed antibiotic growth promoters throughout the whole experimental trial.

## INTRODUCTION

Poultry meat is the most consumed source of animal protein in the world (FAO, 2023) and plays a critical role in satisfying the worldwide growing demand of protein (Van Boeckel et al., 2015). The widespread utilization of antibiotic growth promoters **(AGP)** has been a common practice in poultry to maintain animal health and enhance efficiency (Broom, 2018; Grant et al., 2018). However, AGP may also promote the development of antimicrobial resistances **(AMR)** and become a threat to food safety for consumers. For this reason, the European Union **(EU)** banned their use in poultry diets (EU Regulation 1831/2003, 2003), and this resulted in increased prevalence of foodborne illness-causing bacteria, such as *Salmonella* spp. or *Campylobacter* spp. (Chantziaras et al., 2014).

Probiotic bacteria have emerged as potential alternatives for AGP, for their strain specific potential modes of action such as the control of gut microbiota populations and colonization by pathogens, and the improvement of performance and intestinal health (Cazorla et al., 2018; Tarradas et al., 2020; Tous et al., 2022). In vivo administration of live probiotic microorganisms may improve microbial diversity in the intestine, and promote beneficial bacteria, pathogen exclusion, and immune modulation and detoxification (Stanley et al., 2015; Oakley and Kogut, 2016; Cazorla et al., 2018; Sureshkumar et al., 2021). Moreover, they may also improve relevant intestinal functions such as the secretion of digestive enzymes, nutrients uptake and short chain fatty acids **(SCFA)** production, resulting in better growth performance and feed conversion efficiency (Khalighi et al., 2016; Shokryazdan et al., 2017; Cheng et al., 2021). Specifically, *Lactobacillus salivarius* CTC2197 has been reported to reduce the colonization of *Salmonella enterica* Enteritidis *C-114* in chickens (Pascual et al., 1999) and the commercial blend CF3 product has also been described to have immunomodulatory effects and to reduce the presence of pathogens by competitive exclusion (Corrier et al., 1995; Corrier et al., 1998; Nisbet, 2002). However, their modes of action on host and microbiota are still to be fully deciphered.

On the other hand, it is well documented that diets rich in soluble non-starch polysaccharides **(NSP)** induce mild inflammation in the digestive tract, increase intestinal viscosity, and negatively affect nutrient digestibility and performance in poultry (Choct et al., 2010; Bach Knudsen, 2014; Nguyen et al., 2021; Kim et al., 2022b). The administration of diets rich in soluble NSP without enzymes can be used as a challenge model to characterize the immunomodulatory effects of probiotics in counteracting inflammatory processes in the intestine (Tarradas et al., 2020; Tous et al., 2022). In addition, it has been demonstrated that probiotics can partially mitigate NSP-induced disturbances in nutrient digestibility and absorption (Goodarzi Boroojeni et al., 2024).

To address the existing gaps in knowledge and confront pivotal challenges in poultry production, we conducted 2 in vivo trials to test 3 probiotic strains in broiler chickens fed NSP-rich diets. The probiotic strains tested were *Enterococcus faecalis* SV1028 and *Bacteroides fragilis* GP1764, isolated from a commercial CF3 product, and *Ligilactobacillus salivarius* CTC2197. The aim of these 2 studies was to evaluate the effects of the probiotics (administered individually or as a blend) on performance, intestinal health and mucosal immunity, and gut microbiota of broiler chickens.

## MATERIALS AND METHODS

### Ethics Statement

This study followed the EU principles and according to Directive 2010/63/EU of 22 September 2010, and the Spanish guidelines for the care and use of animals in research (Real Decreto 53/2013). The experimental procedures were approved by the Ethical Committee of Generalitat de Catalunya, Spain (Project number 11697).

### Birds’ Housing and Management

The current study included 2 different trials with broiler chicks Ross308® from a commercial hatchery in different experimental facilities and time periods. In Trial 1, a total of 864 day-of-hatch male broiler chicks (average BW 47.8 ± 0.3g) were allocated in a barn with 24 pens, at 36 birds per pen (free surface of 2.25 m^2^). In Trial 2, a total of 1,040 day-of-hatch male broiler chicks (average BW 39.8 ± 0.6g) were allocated in a barn with 52 pens, at 20 birds per pen (free surface of 1.47 m^2^). Both barns were windowless and provided with automatic environment control with a gas heating system by screens and ventilation by depression, and with programmable lighting. The temperature was adjusted according to the following program: 0 to 2 d → 32 °C to 34 °C; 3 to 7 d → 29 °C to 31 °C; and decreasing 3 °C per week afterwards until reaching 21 °C. The lighting program was 24 h of light the first 2 d, 18 h until 7 d, and 14 h per day afterwards. The litter used was fresh wood shavings. Broilers were vaccinated against Avian Infectious Bronchitis and Gumboro at hatch.

Chicks were counted and weighed by hatchery tray upon arrival (0 d). At 7 (Trial 2) or 8 (Trial 1), 21, and 35 d, birds and feed consumed were weighed per replicate. Growth performance parameters (body weight **(BW)**, average daily gain **(ADG)**, average daily feed intake **(ADFI)**, feed conversion ratio **(FCR)** and European production efficiency factor **(EPEF)** were calculated per each period and for the whole experiment. The EPEF was calculated as follows: EPEF=ADG(g)·[1−(mortality(%)/100]/FCR·10.

Dead birds were weighed, and the most probable cause of death was recorded. Birds (laggards) excluded from the trial during the first week were not considered for performance traits.

On 7 and 21d, 2 birds per pen in Trial 1 (n = 12 per treatment and day) and one bird per pen in Trial 2 (n = 13 per treatment and day) were euthanized and sampled. These chicks were randomly selected and marked at 0 d to avoid observer biases in subsequent samplings. Two fragments of 0.5 × 0.5 cm of jejunum and cecum tissues were collected and stored at −80°C for sIgA analysis. A section of ileum 5 cm near to ileo-cecal junction was collected, fixed in 4% (V/V) phosphate-buffered formaldehyde immediately after sampling for histomorphological analyses and stored at room temperature. Approximately 3g of jejunal and cecal contents were frozen immediately in liquid nitrogen and preserved at -80°C for microbiota and SCFA analysis.

### Diets

Wheat/maize, soybean and rye-based basal diets were formulated to meet or exceed FEDNA recommendations (FEDNA, 2018) in 3 different phases: starter (0–8 d Trial 1, 0–7 d Trial 2), grower (8 or 7 to 21 d for Trial 1 and 2, respectively) and finisher (21–35 d). Feeds were presented in crumble form for all periods. Diets were formulated to induce a moderate (Trial 1) or mild (Trial 2) inflammation in the upper digestive tract through increasing the percentage of soluble NSP (Choct et al., 2010; Bach Knudsen, 2014; Kim et al., 2022c) and without adding exogenous enzymes. The ingredient and calculated nutrient compositions of the basal diets are summarized in Table 1. The basal diets did not contain coccidiostats, antibiotics or enzymes. Feed and water were provided ad libitum consumption.

**Table 1:** Ingredient and nutrient composition of the basal diets (as fed basis).

	Trial 1			Trial 2		
Period (days)	0–8	8–21	21–35	0–7	7–21	21–35
**Ingredient (%)**						
Wheat	48.17	48.16	47.33	25.00	25.00	25.00
Maize	-	-	-	21.74	26.39	28.66
Rye	10.00	12.50	15.00	10.00	10.00	10.00
Extruded soyabean	1.35	9.33	17.53	-	3.13	10.02
Soybean 48%	32.54	22.48	12.99	35.73	27.87	19.06
Animal fat^1^	4.00	4.00	4.00	3.63	4.00	4.00
L-lysine HCL	0.26	0.21	0.20	0.21	0.20	0.20
L-threonine	0.15	0.09	0.08	0.13	0.09	0.08
DL-methionine	0.34	0.27	0.23	0.35	0.29	0.25
L-Valine	0.02	0.02	0.02	-	0.03	0.03
L-tryptophan	0.01	-	-	0.01	-	-
Choline chloride	0.02	0.02	0.02	0.02	0.02	0.03
Sodium chloride	0.37	0.22	0.21	0.38	0.24	0.23
Calcium carbonate	0.47	0.40	0.34	0.44	0.38	0.32
Dicalcium phosphate	1.90	1.67	1.43	1.94	1.73	1.51
Sodium bicarbonate	-	0.20	0.20	-	0.20	0.20
Antioxidant^2^	0.02	0.02	0.02	0.02	0.02	0.02
Vitamin-Mineral premix^3^	0.40	0.40	0.40	0.40	0.40	0.40
**Estimated nutrient contents (%)**						
Metabolizable Energy (Kcal/kg)	2900	3000	3100	2900	3000	3100
Dry Matter	87.86	88.07	88.49	87.14	87.15	87.48
Crude Protein	22.55	20.85	19.48	22.15	19.87	18.36
Crude fiber	2.87	2.76	2.66	2.26	2.23	2.27
Ether extract	5.82	7.21	8.64	5.36	6.40	7.69
Ash	6.01	5.63	5.21	6.03	5.57	5.12
Lys AID^4^	1.25	1.10	1.00	1.25	1.10	1.00
Met AID^4^	0.62	0.53	0.47	0.63	0.55	0.49
Total NSP^5^	14.62	18.02	21.59	13.75	14.54	17.34
Soluble NSP^5^	3.61	4.09	4.61	3.29	3.27	3.62
Insoluble NSP^5^	11.01	13.93	16.97	10.46	11.28	13.72
**Analyzed nutrients (%)**						
Gross energy (Kcal/kg)	4176	4271	4360	4173	4204	4346
Dry matter	89.70	90.18	90.38	89.77	89.50	89.43
Crude protein	22.52	20.74	19.27	22.65	19.99	18.64
Crude fiber	3.02	2.87	2.93	2.72	2.62	2.45
Ether extract	5.65	7.34	8.74	5.32	6.47	8.04
Ash	5.29	4.75	4.09	4.61	4.25	2.79

1 5 Sysfeed (Sysfeed SLU; Granollers, Spain). It contains myristic acid (C14:0) 1.50%, palmitic acid (C16:0) 18.0%, palmitoleic acid (C16:1 n-7) 2.00%, stearic acid (C18:0) 14.0%, oleic acid (C18:1 n-9 cis) 28.0%, linoleic acid (C18:2 n-6 cis) 12.0%, α-linolenic acid (C18:3 n-3 cis) 6.00%, saturated–unsaturated 0.7%.

2 Noxyfeed 56P (ITPSA; Barcelona, Spain). It contains 56% of antioxidant substances (butylated hydroxytoluene + propyl gallate) and synergistic (Citric acid 14% + authorized support).

3 Vitamin-Mineral premix (DEX Ibérica S.A.; Vila-Seca, Spain). Supplied per kilogram of feed: Vitamin A (3a672a retinyl acetate): 10,000 IU; Vitamin D3: 4,800 IU; Vitamin E (3a700α-tocopheryl acetate): 67.2 IU; Vitamin K3: 3 mg; Vitamin B1: 3 mg; Vitamin B_2_: 9 mg; Vitamin B_6_: 4.5 mg: Vitamin B_12_: 40 ug; Folic acid: 1.8 mg; Biotin: 150 ug; Calcium pantothenate: 16.5 mg; Niacin: 65 mg; Mn (as MnSO_4_.H_2_O): 90 mg; Zn (as ZnO): 66 mg; I (as KI): 1.2 mg; Fe (as FeSO_4_.H_2_O): 54 mg; Cu (as CuSO_4_.5H_2_0): 12 mg; Se (as NaSeO_3_): 0.18 mg; BHT: 25 mg; Calcium formiate, 5 mg; Silicic acid, dry and precipitated, 25 mg; Calcium stearate, 25 mg; Calcium carbonate to 4 g.

4 Apparent ileal digestibility.

5 Non-starch polysaccharides, calculated according to (Bach Knudsen, 2014).

The contents of crude protein, total fat, crude fiber, and gross energy of feeds were determined by NIR spectrophotometry, using prediction models that fulfil all the quality requirements stated in ISO 12099:2017 “Animal feeding stuffs, cereals and milled cereal products — Guidelines for the application of near infrared spectrometry”, as follows (Parameter & Reference method AOAC 2000 [Prediction model R2 / Standard Error of Prediction / Uncertainty (±2×SEP)): Crude protein (%) 968.06 [0.98/0.30/0.60]; Total fat (%) 920.39 [0.98/0.19/0.38]; Crude fiber (%) 962.09 [0.96/0.24/0.48]; Gross Energy (kcal/kg) DIN 51900 [0.94/33/66]; dry matter and ash were determined by the AOAC methods 925.09 and 942.05, respectively.

### Probiotics Administration

Every morning, different broth mixtures (in Buffered Peptone Water) were prepared, as required (control and different probiotic treatments), so they could be provided in 5 mL to each experimental pen. That amount of broth was applied on top of a small amount of feed that was provided on its own in a feed pan when the light was switched on. Simultaneously, access to the conventional feeders was restricted until the birds had consumed the experimental feeds. The amount of experimental feed varied with age and between experiments as follows: 1st experiment: 4 g/chick (0–8 d), 8 g/chick (9–21 d) and 12 g/chick (22–35 d); 2nd experiment: 6 g/chick (0–7 d), 12 g/chick (8–21 d) and 24 g/chick (22–35 d).

### Experimental Design

In Trial 1, birds were randomly distributed to one of the 4 experimental diets: Control (**Ctrl**): Basal diet; *EntF*: Basal diet + *E. faecalis; BacF*: Basal diet + *B. fragilis,* or *LacS*: Basal diet + *L. salivarius* for 35 d. The dosage of each probiotic was 5×10^7^ CFU/chick/day.

In Trial 2, birds were distributed to one of the 4 experimental diets: Ctrl: Basal diet; Antibiotic: Basal diet + Zn-Bacitracin (55ppm); Probiotic 0 to 10d: Basal diet + Blend of *EntF* + *BacF* + *LacS* administered from 0 to 10 d of age (and basal diet from 11 to 35 d); and Probiotic 0 to 35 d: Blend of *EntF* + *BacF* + *LacS* administered from 0 to 35 d of age.

The dosage of *EntF* and *BacF* was 1.6 × 10^8^ CFU/chick/day and the dosage for *LacS* was 1 × 10^7^ CFU/chick/day. For the positive control group, 155 mg/kg of Zn-Bacitracin (55ppm) was added on top in feed in the feed mill.

### Histomorphological Analyses

Ileum tissue samples were dehydrated, cleared with xylene and embedded with paraffin. Serial of 3 μm sections were prepared, mounted on glass slides and stained with Alcian blue-periodic acid Schiff **(AB-PAS)** following manufacturer's protocol (AB- 8GX, Sigma; Schiff's reagent, Merck, Darmstadt, Germany). Five villi from each ileal sample were selected for histomorphological analyses (villus height [**VH**], crypt depth [**CD**], villus width [**VW**], and villus height and crypt depth ratio [**V:C**]). The total number of goblet cells (**GC**) in each villus was also counted, and its density calculated from the number of GC per villus divided by 100 μm. All measurements were performed with an Olympus light microscope (BX 43, Olympus, Germany) with a digital camera (DP72, Olympus, Germany). Image analysis was performed by using cellSens Standard software (version 1.14, Olympus, Germany) and ImageJ software (Rasband, W.S. ImageJ, United States National Institute of Health, Bethesda, Maryland, United States).

### Secretory Immunoglobulin A

Chicken specific secretory Immunoglobulin A **(sIgA)** concentrations were determined in cecum and jejunum tissues homogenates according to manufacturer protocol (sIgA ELISA kit catalogue number CEA641Ga - Clone Cloud [Katy, TX]). Briefly, approximately 150 mg of tissue was taken and homogenized in 500 µL of PBS for 1 min with an Ultra Turrax homogenizer system. Cells rupturing was performed 5 times in 15 s intervals by sonication in ice bath with a holding time of 45 seconds. Finally, samples were centrifuged for 15 min at 6,500 rpm and 4°C, and the supernatant was collected and stored at -80°C until sIgA analysis.

### Intestinal Viscosity

After starter phase, and in all chicks of both trials, the intestinal viscosity was assessed, quantifying the number of birds per pen with sticky droppings attached to the vent (analyzed as a percentage of chicks with wet excreta sticking to the down of the cloaca per pen) according to (Francesch et al., 1994).

### Short-Chain Fatty Acids

The determination of SCFA was done according to (Jouany, 1982). Briefly, 1 g of defrosted content was mixed with 1 mL H_2_O, 2 mL C_4_H_10_O, 0.5 mL HCl 37%, and 0.1 mL internal pattern (0.318 mL 4-methyl valeric acid + 1.5 mL NaOH 5M in 50 ml H_2_O) and homogenized 1 min using vortex. Then, samples were centrifuged 15 min at 1,370 g. 65 µL of supernatant were collected and mixed with 10 µL of MTBSTFA (N-metil-N(tert-butildimetilsilil)-trifluoracetamide) for derivatization, incubated 30 min at 80°C, and analyzed by gas chromatography (Agilent Technologies, Wilmington, DE).

### Microbiota Analysis

#### DNA Extraction and 16S Amplicon Sequencing

DNA was extracted from 0.25g of cecal content samples by using DNeasy 96 PowerSoil Pro Kit (Qiagen, Germany), following the manufacturer's instructions. Transcribed 16S rRNA libraries targeting V1–V3 region from bacterial population, were sequenced by utilizing MiSeq Illumina sequencing platform (Illumina, 2 × 300 bp kit, San Diego, CA) following manufacturer's instructions at Molecular Research DNA (Mr. DNA Lab, Shallowater, TX). For the bacterial libraries the primer set 27F (5′-AGRGTTTGATCMTGGCTCAG–3′) / 519R (5′-GTNTTACNGCGGCKGCTG-3′) was used. The obtained DNA reads were compiled in FASTq files for further bioinformatic processing.

#### Bioinformatics Processing of Amplicon Data

Amplicon sequencing reads were demultiplexed based on library indices using AdapterRemoval (Schubert et al., 2016). Using Cutadapt 1.18 (Martin, 2011), primer locations were identified, and reverse complemented the reads in Reverse-Forward direction to ensure unidirectionality of all sequences. Taxonomic assignment was done by the naive Bayesian classifier method with default settings as implemented in DADA2 (Callahan et al., 2016) in R 3.6.1 (Dessau and Pipper, 2008), against SILVA 16S rRNA gene reference taxonomy database. The initial amplicon sequence variants (**ASVs**) table was generated that were taxonomically annotated. The ASVs that could not be taxonomically identified as Bacteria and with low representation (<0.01% of the total reads per sample) were removed and the output ASV table was used for downstream analyses.

#### Diversity and Compositional Analysis

For the microbiota assessment, diversity analyses and visualization were carried out using Hill numbers as implemented in the R package *hilldiv2* (Alberdi and Gilbert, 2019). Specifically, alpha and beta diversity estimations were conducted using 3 metrics accounting for different diversity components and orders. Richness (neutral Hill number of order of diversity q = 0) only considered presence and absence of bacteria. Neutral diversity refers to the Hill number of q = 1 (equivalent to the Shannon diversity or exponential to the Shannon index), which weighs bacteria according to their relative abundances. Phylogenetic diversity refers to the phylogenetic Hill number of q = 1 (equivalent to Allen's H), which also considers the phylogenetic correlations between bacteria.

### Statistical Analysis

The data were explored to discard possible outliers according to the Kolmogorov-Smirnov test (Massey, 1951). Results were analyzed by 2-way ANOVA (GLIMMIX procedure of SAS software [SAS 9.4; SAS Institute Inc., Cary, NC]) to perform the analysis of the different performance variables. In addition, pair-wise comparison of means (Tukey's T test) was used to determine significance between treatment means (*P* < 0.05) for the type I error. The model included block (location in the experimental room) as a randomized effect, and different treatments and their interaction as main effects.

The statistical model used was:

$$y_{\text{ij}}=\mu +\tau_{i}+\gamma_{j}+e_{\text{ij}}$$

Where y_ij_ is the response variable, *τ*_i_ is the dietary treatment effect, *γ*_j_ is the random block effect, and e_ij_ is the error of the experimental unit. The experimental unit for all traits assessed was the pen. Results are expressed as least square means ± standard error of mean.

In the case of ELISA determinations, when the limit of detection was not reached, the missing values were replaced by the limit of detection (LD)/√2 (Hornung and Laurence, 1990). Mortality and culled chicks were assessed using categorical data analysis. Values were expressed as percentages and analyzed non parametrically using Wilcoxon's test.

Microbiota diversity comparisons between treatments were carried out using the Kruskal–Wallis (**K-W**) rank sum test, followed by a posthoc Dunn's test with Bonferroni-corrected p values. Using the adonis function as implemented in the vegan package, compositional differences were compared using permutational multivariate analyses of variance (PERMANOVA) with 999 permutations (Oksanen, 2010). Calc_pairwise_permanovas function of R package mctoolsr was used to calculate pairwise post-hoc comparisons between samples (Leff, 2017). Differences were considered significant at *P* < 0.05, while those at *P* < 0.10 are reported as tendencies.

## RESULTS

### Probiotics Effects on Broilers’ Growth Performance

The results of growth performance are presented in Tables 2 and 3 for Trial 1 and 2, respectively. In Trial 1, single strain probiotic *EntF* increased BW, ADG, ADFI and EPEF and reduced FCR at 8 d compared to birds in control group (*P* = 0.011) although this effect did not persist in later stages of life. *BacF* treatment decreased ADFI of chicks at 8 d (*P* < 0.001) and EPEF from 8 to 21 d of age (*P* = 0.008). On the other hand, *LacS* strain showed a reduced FCR at 8 d compared to birds in control group (*P* = 0.010). However, birds fed this bacterial strain had lower BW from 21 d (*P ≤* 0.025), lower ADG from 8 to 21 d and considering all trial duration (*P ≤* 0.021) and a lower EPEF from 0 to 35 d (*P =* 0.025).

**Table 2:** Effects of probiotics administration on performance traits of broilers (Trial 1)^1^.

	N	BW (g)	ADG (g)	ADFI (g)	FCR (g/g)	EPEF
*0 to 8 d*						
Control	6	208^b^	20.0^b^	23.5^ab^	1.177^a^	170^b^
*EntF*	6	215^a^	20.9^a^	23.8^a^	1.139^b^	183^a^
*BacF*	6	208^b^	20.1^b^	23.0^c^	1.148^ab^	175^ab^
*LacS*	6	211^ab^	20.4^ab^	23.3^bc^	1.140^b^	179^ab^
SEM		1.50	0.18	0.15	0.008	2.60
*P-value*		*0.011*	*0.008*	*<0.001*	*0.010*	*0.011*
*8 to 21 d*						
Control	6	965^a^	58.2^a^	79.2	1.361	426^a^
*EntF*	6	951^ab^	56.7^ab^	79.7	1.409	396^ab^
*BacF*	6	926^ab^	55.2^ab^	77.7	1.409	392^b^
*LacS*	6	914^b^	54.1^b^	75.8	1.402	386^b^
SEM		12.30	0.88	1.34	0.01	8.80
*P-value*		*0.025*	*0.016*	*0.189*	*0.113*	*0.008*
*21 to 35 d*						
Control	6	1985^a^	72.9	117.0	1.608	455
*EntF*	6	1955^ab^	71.7	118.6	1.661	429
*BacF*	6	1885^ab^	68.6	110.9	1.619	422
*LacS*	6	1859^b^	67.5	108.5	1.608	416
SEM		29.20	1.75	2.60	0.03	17.1
*P-value*		*0.021*	*0.109*	*0.050*	*0.370*	*0.226*
*0 to 35 d*						
Control	6	-	55.4^a^	81.6	1.474	374^a^
*EntF*	6	-	54.5^ab^	82.4	1.513	349^ab^
*BacF*	6	-	52.5^ab^	78.5	1.495	350^ab^
*LacS*	6	-	51.7^b^	76.8	1.483	346^b^
SEM		-	0.83	1.49	0.018	7.20
*P-value*		*-*	*0.021*	*0.050*	*0.307*	*0.025*

1 Values are presented as least squares means.

a-b Values within a column without a common superscript differ *P < 0.05*. BW: Body weight; ADG: Average daily gain; ADFI; average daily feed intake; FCR: feed conversion ratio; EPEF: European production efficiency factor.

**Table 3:** Effects of early or long-term probiotics administration on performance traits of broilers (Trial 2)^1^.

	N	BW (g)	ADG (g)	ADFI (g)	FCR (g/g)	EPEF
*0 to 7 d*						
Control	13	186^b^	20.9^b^	22.1^b^	1.060	197^b^
Antibiotic	13	193^a^	21.9^a^	23.1^a^	1.052	209^a^
Probiotic 0-10d	13	189^ab^	21.4^b^	22.4^b^	1.048	204^ab^
Probiotic 0-35d	13	188^b^	21.3^b^	22.4^b^	1.055	202^ab^
SEM		1.90	0.26	0.21	0.01	3.50
*P-value*		*<0.001*	*<0.001*	*<0.001*	*0.281*	*0.001*
*7 to 21 d*						
Control	13	867	48.7	70.5	1.449^a^	331
Antibiotic	13	894	50.1	71.9	1.437^ab^	343
Probiotic 0-10d	13	873	48.8	69.9	1.432^ab^	337
Probiotic 0-35d	13	871	48.8	69.6	1.428^b^	338
SEM		10.90	0.73	0.97	0.06	6.00
*P-value*		*0.071*	*0.195*	*0.079*	*0.036*	*0.456*
*21 to 35 d*						
Control	13	1933	76.2	134.9	1.772^a^	429
Antibiotic	13	1972	77.0	132.7	1.725^b^	445
Probiotic 0-10d	13	1949	76.9	132.8	1.728^b^	442
Probiotic 0-35d	13	1927	75.4	130.6	1.734^ab^	434
SEM		22.90	1.07	1.59	0.01	8.30
*P-value*		*0.235*	*0.505*	*0.108*	*0.007*	*0.259*
*0 to 35 d*						
Control	13	-	54.1	85.9	1.587^a^	335
Antibiotic	13	-	55.2	85.8	1.554^b^	349
Probiotic 0-10d	13	-	54.6	84.9	1.556^b^	344
Probiotic 0-35d	13	-	53.9	83.9	1.556^b^	341
SEM		-	0.65	0.98	0.006	5.40
*P-value*		*-*	*0.234*	*0.152*	*<0.001*	*0.277*

1 Values are presented as least squares means.

a-b Values within a column, without a common superscript differ *P < 0.05*. BW: Body weight; ADG: Average daily gain; ADFI; average daily feed intake; FCR: feed conversion ratio; EPEF: European production efficiency factor.

In the Trial 2, AGP significantly increased BW, ADG, ADFI and EPEF compared to Control during the starter phase (*P* < 0.001). Chicks treated with the blend of probiotics also increased these parameters but only numerically in the same period. The group of probiotics fed 0 to 35 d improved FCR during grower phase (*P* = 0.036) and the group fed probiotics 0 to 10 d improve it during the finisher phase (*P* < 0.007). Taken the whole study period (0 to 35 d), both Probiotics groups improved FCR at the same extent as Antibiotic compared to Control group (*P* < 0.001).

### Probiotics Effects on Viscosity of Intestinal Content

In Trial 1, there was a high percentage of chicks with sticky droppings in the vent in all groups at 7d (it ranged between 11.9 and 26.5 %; Table 4). In this study, *LacS* increased viscosity of intestinal content compared to control and to the other 2 probiotic groups (*P* = 0.004). In Trial 2, the percentage of sticky droppings at 7d was lower (it ranged between 2% and 5%), and no differences versus the control group were observed for the groups treated with probiotics or AGP.

**Table 4:** Effects of probiotic administration on sticky droppings of birds after starter period^1^.

Sticky droppings (%)	
*Trial 1*	
Control	13.4^b^
*EntF*	12.3^b^
*BacF*	11.9^b^
*LacS*	26.5^a^
SEM	2.72
*P-value*	*0.004*
*Trial 2*	
Control	2.0
Antibiotic	5.4
Probiotic 0–10d	2.7
Probiotic 0–35d	3.1
SEM	1.34
*P-value*	*0.304*

1 Values are presented as least squares means.

a-b Values within a column without a common superscript differ *P* < 0.05.

### Probiotics Treatment Effects on Ileal Morphology

In Trial 1, no significant differences were observed in ileal morphological measurements in response to different individual probiotics administration at 7 d (Table 5). However, *LacS* decreased VH compared to the Control group at 21 d (*P* = 0.024). Nevertheless, no significant differences were observed at 21 d for CD, VW and GC count among Control and the different probiotics. In Trial 2, no significant effects were observed on the intestinal morphology traits assessed in the ileum after the administration of Probiotics blend or Antibiotic compared with control group (Table 5). However, birds fed the antibiotic diet had wider villi compared to those fed the probiotic blend from 0 to 10 d at 7 d.

**Table 5:** Effects of probiotics administration on ileal morphology (Trial 1 and Trial 2).^1^

	VH (µm)	CD (µm)	VW (µm)	VH/CD	GC	GC/100µm
*Trial 1*						
*Day 7*						
Control	473.1	163.1	158.0	2.9	66.7	14.1
*EntF*	498.8	163.9	167.8	3.2	70.9	14.3
*BacF*	450.4	158.1	173.1	2.9	64.3	14.3
*LacS*	437.9	161.8	163.7	2.8	69.0	15.8
SEM	20.01	10.08	7.65	0.20	4.36	0.86
*P-value*	*0.192*	*0.968*	*0.523*	*0.464*	*0.737*	*0.471*
*Day 21*						
Control	665.0^a^	175.7	165.3	3.8	111.7	16.9
*EntF*	657.0^ab^	187.8	183.3	3.5	98.3	15.0
*BacF*	637.1^ab^	171.1	157.0	3.7	106.3	16.8
*LacS*	603.8^b^	162.1	148.0	3.8	98.7	16.3
SEM	14.29	7.48	12.20	0.18	5.56	0.90
*P-value*	*0.024*	*0.150*	*0.251*	*0.607*	*0.268*	*0.364*
*Trial 2*						
*Day 7*						
Control	183.1	64.4	66.4^ab^	2.9	77.3	42.4
Antibiotic	202.2	60.8	67.0^a^	3.3	85.9	43.1
Probiotic 0-10d	169.6	57.1	58.4^b^	3.0	76.1	45.0
Probiotic 0-35d	195.8	59.1	61.2^ab^	3.3	84.6	43.9
SEM	21.86	5.23	2.46	0.25	9.47	2.43
*P-value*	*0.634*	*0.693*	*0.027*	*0.661*	*0.831*	*0.894*
*Day 21*						
Control	306.3	81.4	75.3	3.9	135.8	44.4
Antibiotic	299.0	80.1	83.2	3.8	143.3	47.9
Probiotic 0-10d	285.0	76.8	77.0	3.7	144.3	50.3
Probiotic 0-35d	344.9	87.4	77.2	3.9	138.8	41.7
SEM	24.95	6.46	4.44	0.24	12.97	3.03
*P-value*	*0.311*	*0.650*	*0.504*	*0.851*	*0.950*	*0.230*

1 Values are presented as least squares means.

a-b Values within a column without a common superscript differ *P < 0.05*. VH: Villus height; CD: Crypt depth; VW: Villus width; VH/CD: Villus height to Crypt depth ratio; GC: Goblet cell.

### Influence of Probiotics on Secretory IgA Levels in Jejunum and Cecum Tissues

In Trial 1, there were no significant differences in jejunum and cecum on the sIgA concentrations among the probiotics fed groups and Control at 7 d (Table 6). However, sIgA concentration in jejunum was significantly higher in *BacF* compared to *LacS* probiotic group at 21 d (*P* = 0.048).

**Table 6:** Effects of probiotics administration on Secretory IgA concentration^1^.

	Jejunum	Cecum
***Trial 1***		
***Day 7***		
Control	5.30	5.01
*EntF*	5.31	5.23
*BacF*	5.41	5.16
*LacS*	5.41	5.31
SEM	0.07	0.18
*P-value*	*0.561*	*0.711*
***Day 21***		
Control	6.69^ab^	7.07
*EntF*	6.52^ab^	6.92
*BacF*	7.04^a^	7.09
*LacS*	6.43^b^	6.83
SEM	0.16	0.20
*P-value*	*0.048*	*0.748*
***Trial 2***		
***Day 7***		
Control	4.76	4.75^a^
Antibiotic	4.83	4.58^b^
Probiotic 0–10d	4.84	4.78^a^
Probiotic 0–35d	4.80	4.75^a^
SEM	0.05	0.04
*P-value*	*0.631*	*0.009*
***Day 21***		
Control	5.20	5.07
Antibiotic	5.14	4.95
Probiotic 0–10d	5.17	5.03
Probiotic 0–35d	5.18	5.01
SEM	0.07	0.06
*P-value*	*0.954*	*0.327*

1 Values (Log_2_ pg/mL) are presented as least squares means.

a-b Values within a column without a common superscript differ *P < 0.05*.

In the Trial 2, Antibiotic decreased sIgA concentration in cecum at 7 d compared to Control and Probiotics feed diet group (*P =* 0.009) while no significant differences were observed at 21 d (Table 6). Similarly, no significant differences with the control were observed in jejunum at 7 or 21 d in response to the Antibiotic or Probiotics.

### Probiotics Effects on Cecal Microbiota Diversity and Composition

In Trial 1, alpha diversity differences were observed in all the experimental groups in terms of richness and only in control and *LacS* groups in terms of neutral, from 7 d to 21 d (Table S1).

**Figure 1:**
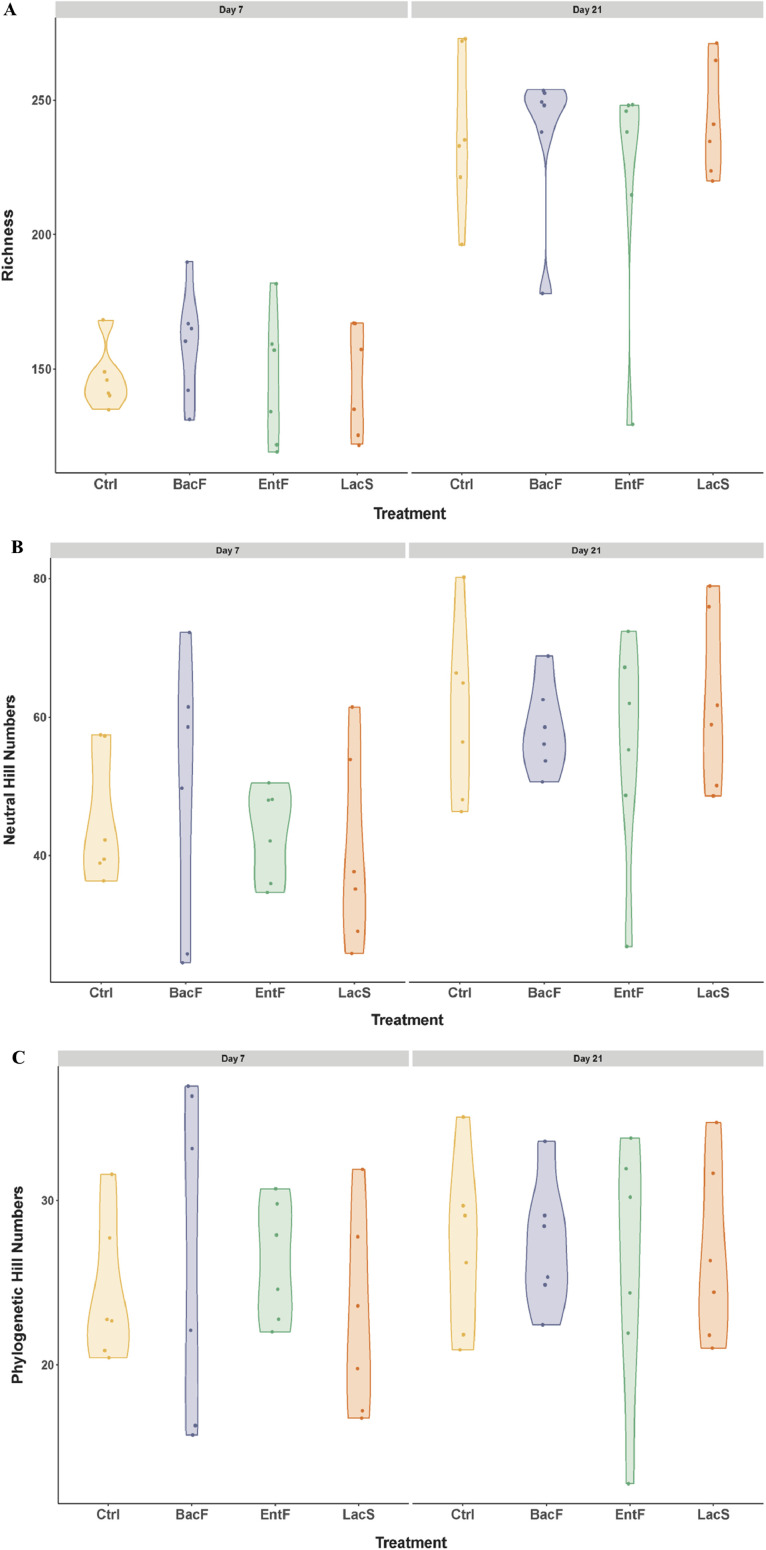
Effects of probiotics administration on cecal alpha diversity plots in Trial 1. (A) richness, (B) neutral and (C) phylogenetic between treatments across time.

**Figure 2:**
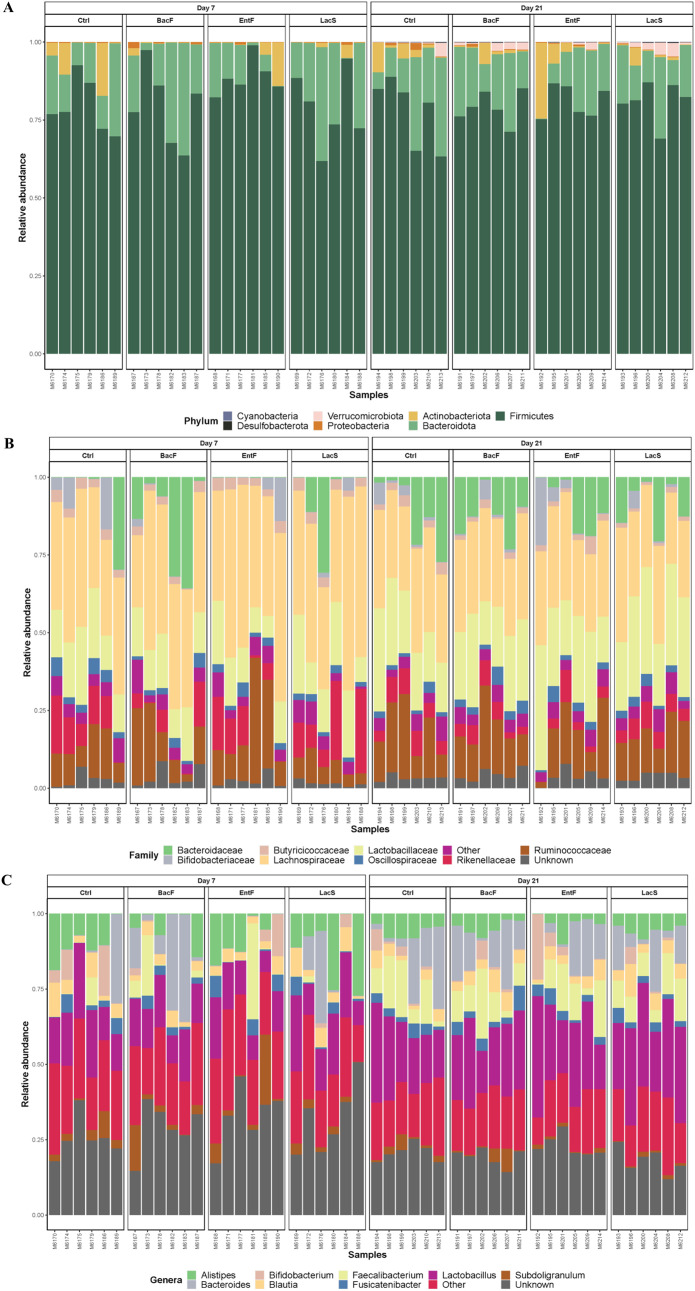
Effects of probiotic administration on taxonomic classification of cecal microbiota across days and treatments in Trial 1. Stacked bar chart of intestinal bacteria by relative abundance (A) at phylum, (B) family and (C) genera level.

There wasn't any difference in alpha diversity indices among treatments (Figure 1), though it was composed mainly of phylum Firmicutes and Bacteriodota, Families *Lactobacillaceae* and *Lachnospiraceae*, and genera *Lactobacillus* (Figure 2).

**Figure 3:**
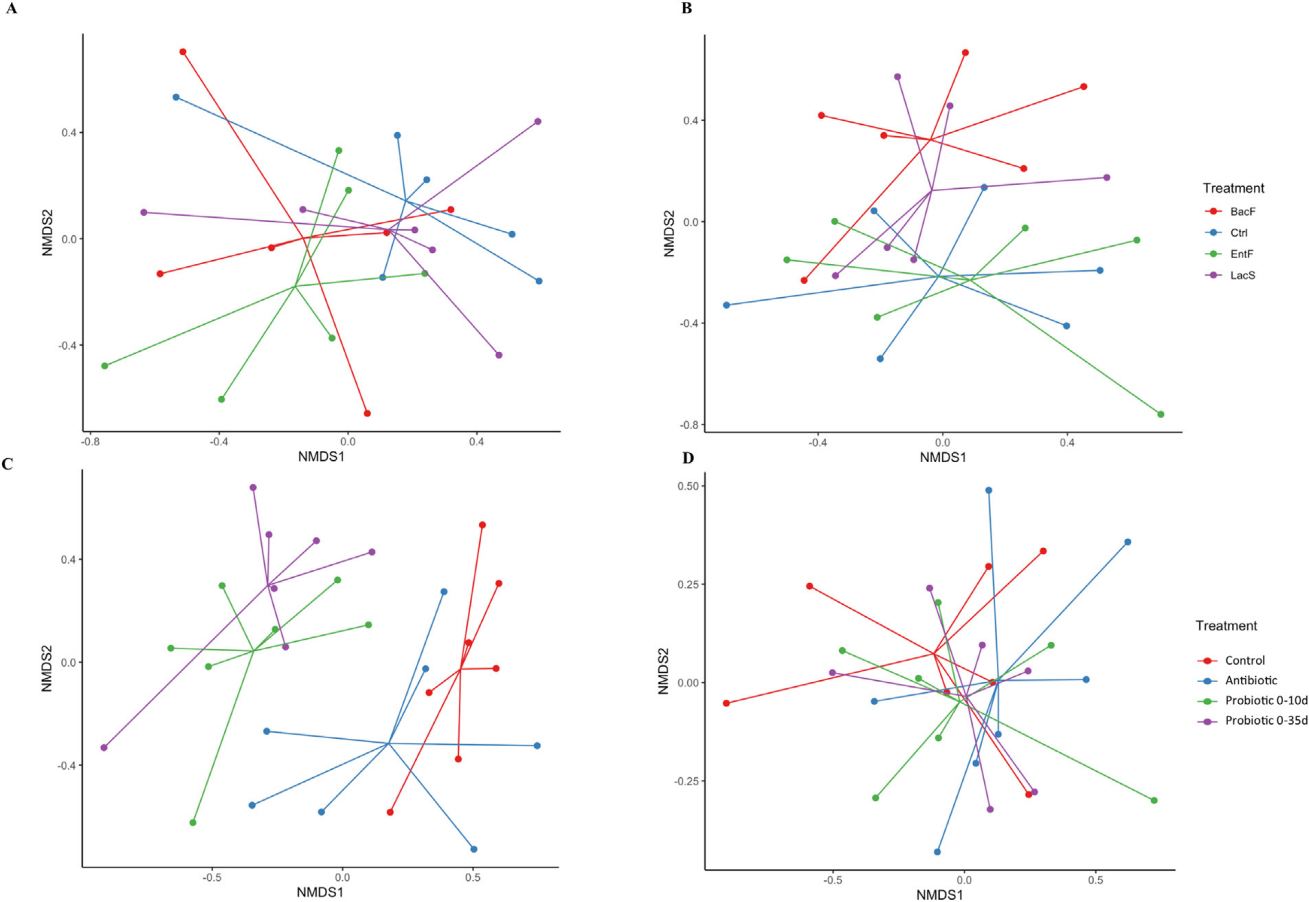
Effects of probiotic administration on cecal microbial Nonmetric multidimensional scaling (NMDS) ordination plot showing community differences among treatments across time in Trial 1; (A) 7 d and (B) 21 d, and Trail 2; (C) 7 d and (D) 21 d.

There was no difference in microbiota composition on 7 d and 21 d (Figures 3A and 3B), however, on 7 d, although not significantly different, the microbiota composition in *EntF* group clustered slightly apart from the rest of the treatments (Figure 3A).

**Figure 4:**
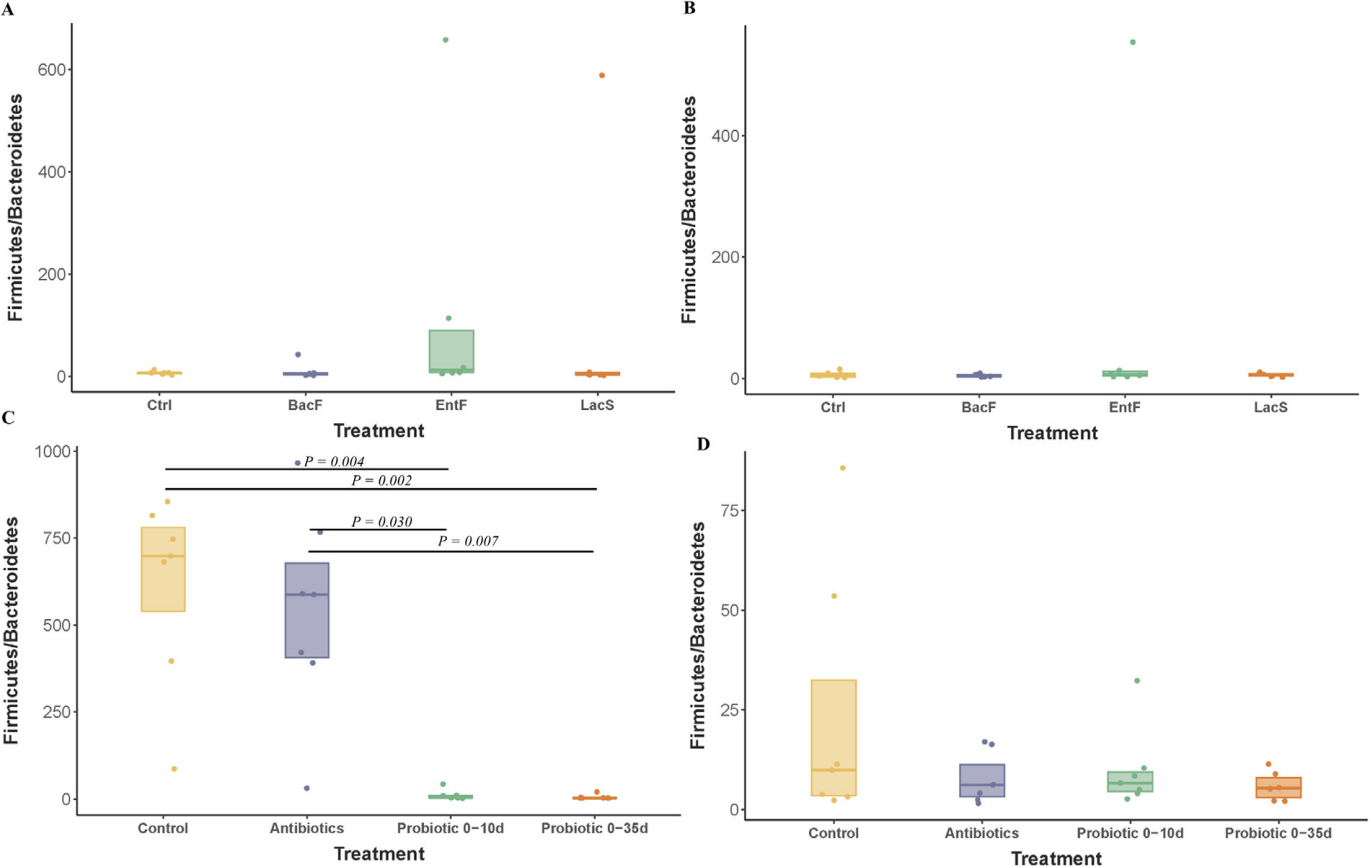
Effects of probiotic administration on cecal *Firmicutes/Bacteroides* ratio in Trial 1; (A) 7 d and (B) 21 d, and Trail 2; (C) 7 d and (D) 21 d.

Richness was higher on 21 d in all treated groups (Table S1), with *LacS* being the highest. Moreover, on 21 d, a higher abundance of genus *Faecalibacterium* and *Lactobacillus* was observed compared to 7 d in all groups (Figure 2C). No differences were observed in Firmicutes to Bacteroidota ratio (**FBR**) at 7 d neither 21 d (Figures 4A and 4B).

**Figure 5:**
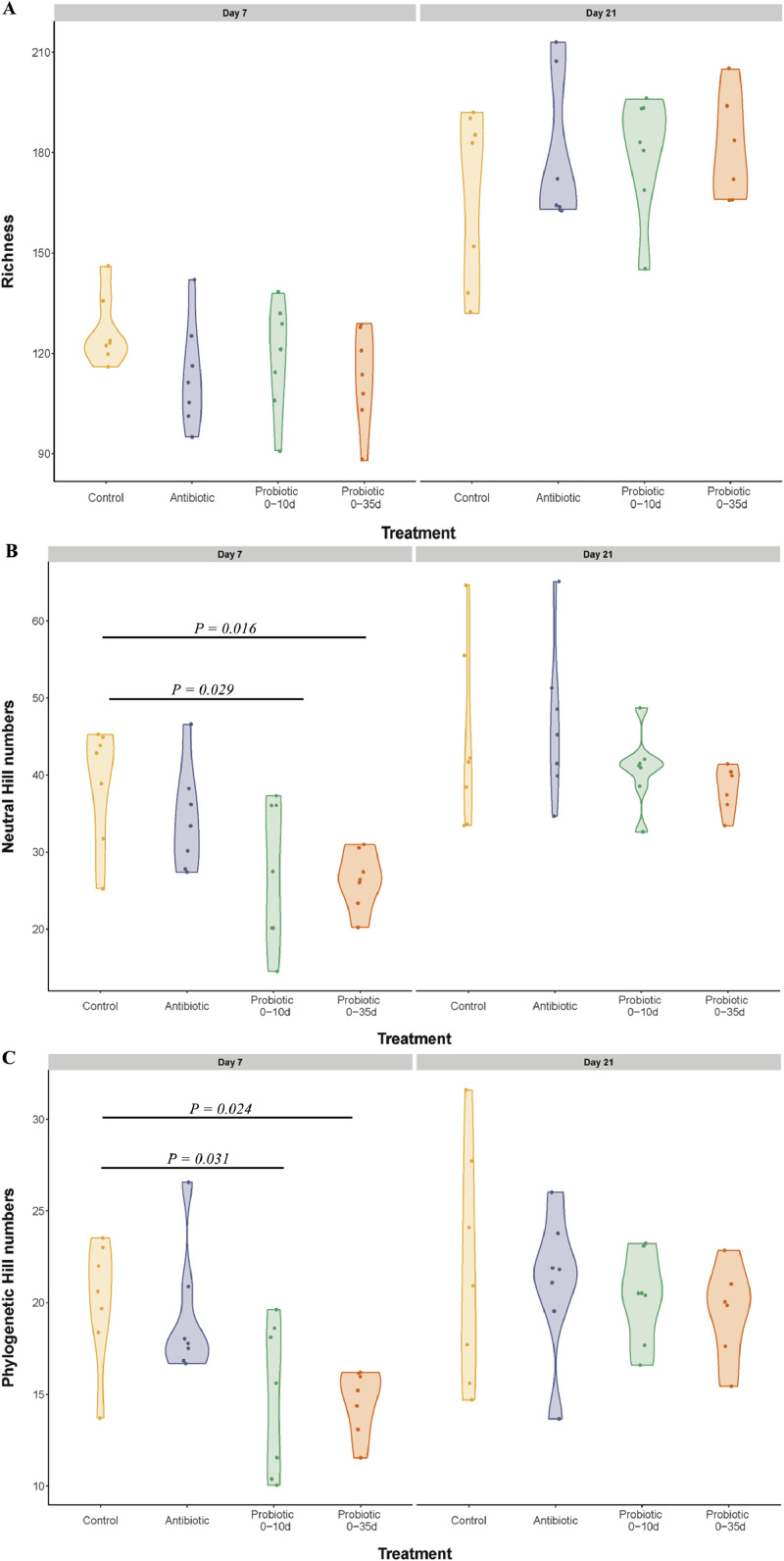
Effects of early probiotics administration on cecal alpha diversity plots in Trial 2. A) richness, B) neutral and C) phylogenetic between treatments across time.

In Trial 2, alpha diversity differences were observed in all the experimental groups in terms of richness and for Probiotics 0-10d group only in terms of neutral, from 7 d to 21 d (Table S1). On 7 d, probiotic-treated chicks from both groups showed a lower neutral and phylogenetic alpha diversity than control chicks (Figures 5B and 5C).

**Figure 6:**
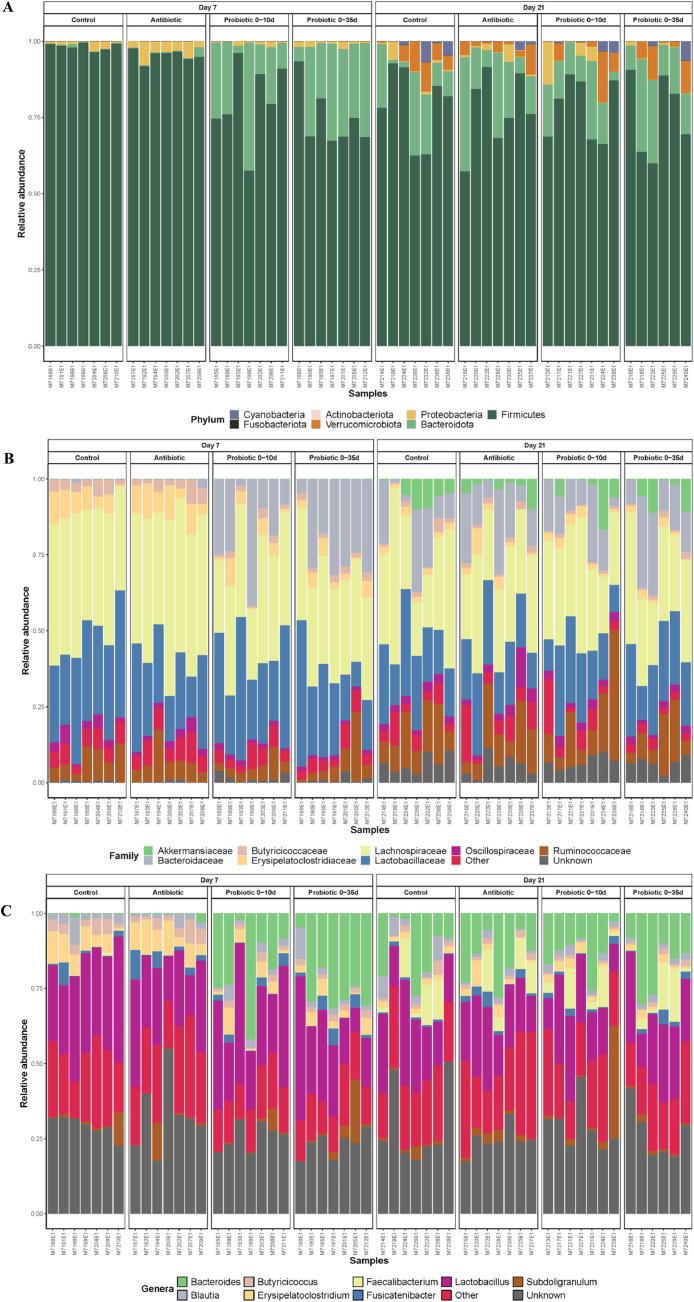
Effects of early probiotics administration on taxonomic classification of cecal microbiota across days and treatments in Trial 2. Stacked bar chart of intestinal bacteria by relative abundance (A) at phylum, B) family and C) genera-level.

At 7 d, the microbiota composition of birds clustered in 2 main groups, the ones fed the Probiotics, and the ones fed the Control or Antibiotic diet (Figure 3C; R^2^ = 0.302, *P* < 0.010). However, these differences disappeared at 21 d (Figure 3 D, R^2^= 0.080, *P* = 0.930). Beta-dispersion analysis showed no differences among all the treatments analyzed in this study across time (*P* > 0.050). Similarly, to the Trial 1, the cecum microbiome of birds at 7 d was composed mainly of phylum *Firmicutes*, Families *Lactobacillaceae* and *Lachnospiraceae*, and genera *Lactobacillus* (Figure 6). At 7 d, the administration of probiotic blend increased the relative abundance of Bacteroidota, specifically the genus Bacteroides (*P* < 0.001). While the FBR of antibiotic-treated chicks was similar to control birds at 7 d (Figure 4C; *P* = 0.700), both probiotic treatments showed significantly lower values compared to the Control and Antibiotic group (Figure 4C). At 21 d, the FBR was similar in all groups (Figure 4D, *P* > 0.050).

### Probiotics Effects on Jejunal and Cecal Short Chain Fatty Acids Concentration

In Trial 1, at 7 d, *EntF* increased acetic acid concentration in cecum compared to *BacF* (*P* = 0.021) but reduced succinic acid concentration in jejunum compared to *LacS* (Table S2, *P* = 0.019). At 21 d, *BacF* increased butyric acid concentration in cecum compared to control birds (*P* = 0.020). In Trial 2, SCFA composition was not affected by the dietary treatment at 7d. However, birds fed the Antibiotic diet had a higher acetic acid concentration in cecum at 21 d compared to Probiotic 0 to 10d group (Table S3, *P* = 0.034). In jejunum, antibiotic and probiotic groups had increased the concentration of lactic acid compared to birds in the control treatment (*P* = 0.010).

## DISCUSSION

This study aims to evaluate the impact of 3 probiotics strains administered individually (Trial 1) or in a blend (Trial 2) on performance, intestinal health and microbiota of broilers. Our investigations focused on examining the influence of *E. faecalis* SV1028*, B. fragilis* GP1764*,* and *L. salivarius* CTC2197 on a range of poultry performance traits and indicators of gut health, namely intestinal morphology, sIgA, microbiota diversity and composition and SCFA in intestinal contents.

It is well known that if chicks grown in optimal conditions, the ability to detect putative beneficial effects of probiotics on birds’ health is reduced (Dal Pont et al., 2021). The use of diets with high concentration of soluble NSPs as a challenge model can be used as a strategy to generate an inflammatory process in the intestine, inducing an ideal environment where the effects of probiotics can be investigated (Kim et al., 2022c; Tous et al., 2022). For this reason, in the current study the birds were challenged by feeding diets containing a high amount of soluble NSPs (Choct et al., 2010) and without exogenous enzymes, aiming to induce a mild or moderate inflammation in the upper digestive tract (Dal Pont et al., 2021; Kim et al., 2022b, c). Our performance results in the negative control groups in both trials showed a reduction of performance compared to broiler performance objectives (Aviagen, 2014), validating the success of our challenge model. These results are in accordance with Kim et al., (2022) which showed that birds fed diets rich in NSP and without exogenous enzymes induced a performance reduction probably due to a hampered nutrient digestibility (Kim et al., 2022a, b, c).

The addition of *E. faecalis* to chicken diets led an increase of BW, ADG, ADFI, and EPEF and a lower FCR suggesting its potential use during the starter phase of broiler chickens. However, these differences gradually decreased throughout the study, possibly because the dose of probiotics per chicken and day was kept constant during the entire experiment. Our results are in accordance with recent studies, which showed a positive effect of *E. faecalis* supplementation on feed efficiency (Park et al., 2016). Other studies observed and improved nutrient digestibility (Zhang et al., 2019) and diverse cecal microbiota while also reducing faecal coliform counts (Shehata et al., 2020) which can be an explanation for the improvement of growth in this study. A higher feed intake was noticed in *B. fragilis* fed group in this study in starter phase and it is in accordance with the studies reporting *Bacteroides* species for its ability to improve nutrient absorption and digestion, fermentation of complex carbohydrates and production of SCFAs (serving as energy source for the host) (Sun et al., 2019; Chen et al., 2022; Wang et al., 2023).

Contrary to the expected, in our study, a lower BW was noticed in the chickens fed *L. salivarius* compared to the control group. This weight loss could be attributed to the biofilm formation capacity of this specific strain (Pascual et al., 1999), causing an increase of viscosity and lower nutrient absorption. This biofilm formation has been related with a reduction in infection and invading capacity of relevant pathogens, such as *Salmonella enterica* serovar Enteritidis, by blocking its adherence and colonization on the epithelial barrier (Garriga et al., 1998; IuV, 2010; Pham et al., 2009). However, an excess of biofilm can increase the viscosity of digesta with a direct effect on the intestinal transit time (Kim et al., 2022c) and, consequently, inducing inflammation in the gut (Dal Pont et al., 2021). In fact, and compared with the negative control, we detected an increase of sticky droppings on the bottom vent of chickens in this group, which are directly related with the increase of viscosity. Moreover, a reduction in the VH was observed in *LacS* group compared with Control indicating a deterioration of gut health (Broom and Kogut, 2018).

The objective of Trial 2 was to determine the effect of the 3 probiotic strains tested in Trial 1 when administered as a blend. In this case, the doses of *E. faecalis* SV1028 and *B. fragilis* GP1764 per chick and day in the blend were increased in respect to Trial 1 to promote the previously detected beneficial effects on performance throughout the growth period. In contrast, *L. salivarius* CTC2197 dose was decreased to control intestinal viscosity and avoid the resulting BW loss observed in Trial 1 but maintaining the beneficial effects on epithelium barrier protection. Furthermore, and taking into account the moderate detrimental effects on performance induced by the challenging diet used in trial 1, the ingredients rich in soluble NSP (wheat and rye) were included in a lower percentage in the Trial 2 (Table 1) to induce a milder inflammatory process in the intestine.

Results from this Trial showed improvements on FCR from 0 to 35d by the administration of the probiotic blend from 0 to 10 d or during the whole experiment similar to the Antibiotic diet. Furthermore, the mixture showed a numerical increase of BW, ADG and ADFI at the same level as of AGP in comparison to negative control group in starter phase, revealing positive effects of probiotic blend on performance of birds.

On the other hand, it is well known that the hydrolysis of NSP through the microbial fermentation generates bioactive substances which can have beneficial effects on gut health through their interactions within the gastrointestinal tract microenvironment (Cisek and Binek, 2014; Svihus and Hervik, 2019; Ćesić et al., 2023). End products of these oligosaccharides may include branched or short-chain fatty acids that can sustain low pH (preventing the growth of pathogenic bacteria) and supply energy to the gut epithelium (Correa-Oliveira et al., 2016; Keerqin et al., 2017; Broom, 2018). Changes in microbial populations can be related to differential concentration of volatile fatty acids in cecum contents (Saleem et al., 2020). In our studies, we detected an increase of butyric acid in cecum when chickens were treated with *B. fragilis* individual probiotic. Moreover, the administration of probiotic blend or antibiotic also increased lactic acid in jejunum at d 21. The increase of this SCFA has been related to an enhanced growth performance and a decreased mortality in poultry (Qiu et al., 2021). It has been previously reported that *B. fragilis* are proven strains to raise aid in the breakdown of complex carbohydrates and support healthier gut barrier function (Sun et al., 2019), and *L. salivarius* promotes the production of short-chain fatty acids, such as lactic acid, creating a less favorable environment for pathogenic bacteria (Sureshkumar et al., 2021; Guerrero Sanchez et al., 2022). Although chickens fed the probiotic blend had positive effects on FCR, in-depth analyses are required to confirm the direct effect of these SCFA variations induced by our probiotic strains on gut health and performance of chickens.

The increased levels of sIgA at 21d in chicks treated with *BacF* (only compared to *LacS*) could be related to a T cell-dependent sIgA response through a noncanonical glycan-dependent binding of sIgA to *B. fragilis.* The heavily N-glycosylated secretory component of sIgA protects the complex from proteolysis and may interact with intestinal bacteria conferring microbiota binding capacities. These sIgA glycan–bacteria interactions might serve as: provision of a carbon source, signals for altering bacterial gene expression or generation of weak inter- or intra-species associations (Pabst and Slack, 2020). In fact, it has been demonstrated that *B. fragilis* is able to efficiently deglycosylate complex N-linked glycans (Cao et al., 2014). Furthermore, in humans, it has been observed that intestinal colonization with *B. fragilis* in early infancy was closely associated with maturation of IgA-secreting cells, confirming that *B. fragilis* is essential for producing IgA (Grönlund et al., 2000; Kumagai et al., 2012). It can be hypothesized that the ability of *B. fragilis* to induce the expression of sIgA could be related to its own needs to be linked with the secretory component of sIgA. In any case, our results are not sufficiently robust to confirm this hypothesis, and specific studies focused on this topic are required to demonstrate the sIgA glycan–bacteria interactions and their benefits for animal health.

Little is known about the modes of action of AGP on host's immune pathways. In our study, compared to the other groups, the addition of AGP to the diet reduced sIgA concentration in cecum at 7d (but not in jejunum). Recently, a study analyzing the effects of bacitracin and colistin in pullets, also obtained no differences in jejunum, but sIgA levels in cecum mucosa were not assessed (Liu et al., 2020). The observed increases of cecal sIgA levels reinforces the hypothesis that AGP may regulate host's homeostatic control mechanisms generating a dynamic equilibrium in the intestine and improve animal health and performance (Fernández Miyakawa et al., 2024). However, more research is needed to understand the direct effects of antibiotics on host when are used as growth promoters.

Regarding the microbiota analyses, the bacterial populations of chickens fed *E. faecalis* SV1028 tended to cluster separately (though not significantly) from the rest of the treatments, probably due to an increase of Firmicutes for its own colonization. Previous studies demonstrated that *E. faecalis* contributed to the improvement of performance traits and to the stability of the microbial community proving to be an important bacterium in maintaining intestinal homeostasis (Zhang et al., 2019; Shehata et al., 2020). Although, the outcomes of the probiotic bacteria used in the present studies may be related to other mode of actions, namely the modulation of gut microbial populations, complementary analyses are required to confirm the direct capacity of the evaluated probiotic strains to degrade NSP.

During the first week after hatch, the microbial composition of chickens treated with the probiotic blend (from 0 to 7 d or from 0 to 35 d) differed from antibiotic-treated and negative control chickens, as a result of a decreased FBR. Chickens treated with the probiotic blend showed a similar FBR at 7 d to that observed at 21 d in all groups, suggesting an early maturation of cecal populations during the first week of life. These results were observed in chickens fed probiotics only 10 d or during all the study, demonstrating that an early administration of probiotics, and only during the first 10 d of life, could accelerate the stabilization of microbial populations during the entire productive cycle of chickens. These results indicate that the first week of life may be an open window to modify the microbiota populations which may have direct implications on performance at the end of the study. Moreover, probiotics modified the microbial communities differently than AGP and control groups but obtaining similar beneficial effects on FCR at 35 d suggesting that the mode of action of probiotics differs from AGP. The microbiota results observed in this Trial are in consonance with the ones observed in other studies (Keerqin et al., 2021), in which the administration of probiotic *Bacillus subtilis* in chickens under a Necrotic Enteritidis’ challenge improved performance parameters and modulated microbiota composition and abundance.

In conclusion, administration of probiotics, *E. faecalis* SV1028 (individually and in starter phase), and *E. faecalis* SV1028*, B. fragilis* GP1764*,* and *L. salivarius* CTC2197 (as a blend) partially improved performance of chickens, counteracting the antinutritional effects induced by a challenging diet rich in NSPs. The probiotic blend administered during the first days’ post-hatch induced similar beneficial effects on FCR compared to antibiotic group or chickens fed probiotic blend during all the trial, demonstrating that the administration of probiotics may not be necessary during all the entire productive life. Furthermore, the shifted microbiota composition observed at 7 d but not at 21 d indicates an opened window to modify the microbiota populations during the first week of life with direct implications on performance at the end of the poultry productive cycle.

## ACCESSION INFORMATION FOR SEQUENCING

For total bacterial populations, data from MiSeq NGS assessment were submitted to the Sequence Read Archive (SRA) of the National Center for Biotechnology Information (NCBI) with the BioProject ID: PRJNA1126084.

## DISCLOSURES

The authors report no conflict of interests.

## Appendix Supplementary materials

Image, application 1

## Footnotes

Supplementary material associated with this article can be found in the online version at doi:10.1016/j.psj.2024.104401.
