# Supplementary material for: Positive impact of early-probiotic administration on performance parameters, intestinal health and microbiota populations in broiler chickens
Source: Poult Sci. 2024 Oct 10;103(12):104401. doi: 10.1016/j.psj.2024.104401 (PMC11566344; doi:10.1016/j.psj.2024.104401)
Supplement: Supplementary file 1 [file mmc1.docx]

EARLY-PROBIOTIC ADMINISTRATION IN BROILERS

**Positive impact of early-probiotic administration on performance parameters, intestinal health and microbiota populations in broiler chickens**

M. Hussain*^,ǁ^, O. Aizpurua^†^, A. Perez de Rozas^‡^, N. París*, M. Guivernau^§^, A. Jofre^#^, N. Tous*, ZW. Ng’ang’a*^,ǁ^, A. Alberdi^†^, E. Rodríguez-Gallego^ǁ^, MH. Kogut^ǂ^, J. Tarradas*^[[1]](#footnote-2)^

**Table S1** Effects of probiotic administration on ceacal microbiota alpha diversity values across time (Trial 1 and Trial 2)^1^.

|  | **Richness** | | | **Neutral** | | | | **Phylogenetic** | | |
| --- | --- | --- | --- | --- | --- | --- | --- | --- | --- | --- |
|  | **Day 7** | **Day 21** | *P-value* | **Day 7** | **Day 21** | | *P-value* | **Day 7** | **Day 21** | *P-value* |
| ***Trial 1*** |  |  |  |  |  | |  |  |  |  |
| Control | 146.5±11.61 | 238.3+29.89 | <0.001 | 45.3+9.55 | | 60.38±12.76 | 0.043 | 24.4±4.40 | 27.14±5.31 | *0.345* |
| *EntF* | 145.5±24.63 | 220.7±46.63 | 0.006 | 43.2±6.74 | | 55.40±16.32 | 0.122 | 26.3±3.69 | 25.84±7.85 | *0.898* |
| *BacF* | 159.2±20.66 | 236.7±29.30 | <0.001 | 48.7±19.63 | | 58.38±06.53 | 0.279 | 26.8±9.90 | 27.30±3.94 | *0.906* |
| *LacS* | 145.5±20.69 | 242.7±21.10 | <0.001 | 40.5±14.14 | | 62.37±12.74 | 0.018 | 22.8±6.09 | 26.67±5.49 | *0.279* |
| ***Trial 2*** |  |  |  |  | |  |  |  |  |  |
| Control | 126.7±10.50 | 167.4±25.90 | 0.003 | 40.0±7.691 | | 44.2±11.67 | 0.906 | 20.1±3.37 | 21.8±6.36 | *0.992* |
| Antibiotic | 113.6±15.96 | 178.0±22.15 | <0.001 | 34.2±6.806 | | 46.6±9.87 | 0.079 | 19.2±3.54 | 21.1±3.88 | *0.978* |
| Probiotics 0-10d | 118.7±16.39 | 180.0±18.03 | <0.001 | 27.4±9.303 | | 40.8±4.77 | 0.042 | 14.8±4.12 | 20.3±2.49 | *0.147* |
| Probiotics 0-35d | 113.0±14.70 | 181.2±16.03 | <0.001 | 26.4±3.807 | | 38.1±3.03 | 0.144 | 14.6±1.78 | 19.5+2.61 | *0.316* |

^1^Values are presented as least square mean ± SD.

**Table S2** Effects of probiotic administration on short chain fatty acid concentration in jejunal and caecal contents (Trial 1).

|  | Formic acid | Acetic acid | Succinic acid | Butyric acid | Isovaleric acid | Valeric acid | Propionic acid | Lactic acid |
| --- | --- | --- | --- | --- | --- | --- | --- | --- |
| ***Day 7*** |  |  |  |  |  |  |  |  |
| ***Jejunum*** |  |  |  |  |  |  |  |  |
| Control | 7.5±1.11 | 1.2±0.32 | 0.3±0.06^ab^ | ND | ND | ND | ND | 8.0±2.18 |
| *EntF* | 7.5±1.21 | 0.2±0.39 | 0.1±0.06^b^ | ND | ND | ND | ND | 7.0±2.54 |
| *BacF* | 7.0±1.10 | 0.3±0.35 | 0.2±0.06^ab^ | ND | ND | ND | ND | 11.2±2.18 |
| *LacS* | 7.1±1.10 | 0.8±0.32 | 0.4±0.06^a^ | ND | ND | ND | ND | 8.2±2.18 |
| *P-value* | *0.983* | *0.132* | *0.019* | - | - | - | - | *0.398* |
| ***Caecum*** |  |  |  |  |  |  |  |  |
| Control | 6.7±1.01 | 8.2±1.52^ab^ | 6.3±1.80 | 2.5±0.74 | ND | ND | ND | ND |
| *EntF* | 7.5±1.13 | 10.9±1.52^a^ | 2.2±1.96 | 3.4±0.74 | ND | ND | ND | ND |
| *BacF* | 5.1±1.03 | 2.9±1.67^b^ | 4.6±1.80 | 0.8±0.81 | ND | ND | ND | ND |
| *LacS* | 7.3±1.03 | 8.9±1.52^ab^ | 5.6±1.80 | 2.3±0.74 | ND | ND | ND | ND |
| *P-value* | *0.389* | *0.021* | *0.367* | *0.173* | - | - | - | - |
| ***Day 21*** |  |  |  |  |  |  |  |  |
| ***Jejunum*** |  |  |  |  |  |  |  |  |
| Control | 1.6±0.97 | 0.2±0.18 | 0.4±0.04 | ND | ND | ND | ND | 8.6±2.12 |
| *EntF* | 2.9±0.90 | 0.2±0.16 | 0.3±0.04 | ND | ND | ND | ND | 10.9±2.12 |
| *BacF* | 1.3±0.97 | 0.7±0.15 | 0.2±0.04 | ND | ND | ND | ND | 5.7±1.94 |
| *LacS* | 1.3±0.97 | 0.2±18 | 0.3±0.04 | ND | ND | ND | ND | 6.8±2.12 |
| *P-value* | *0.493* | *0.078* | *0.360* | - | - | - | - | *0.322* |
| ***Caecum*** |  |  |  |  |  |  |  |  |
| Control | 8.5±2.54 | 25.1±8.21 | 1.7±4.11 | 3.3±1.29^b^ | 0.10±0.06 | 0.5±0.18 | 2.2±2.13 | ND |
| *EntF* | 7.7±2.94 | 33.7±11.61 | 5.1±5.81 | 1.4±1.89^b^ | 0.22±0.09 | 0.9±0.25 | 1.5±3.02 | ND |
| *BacF* | 10.9±2.62 | 46.8±8.99 | 15.8±4.50 | 8.9±1.41^a^ | 0.13±0.08 | 0.5±0.21 | 8.8±2.34 | ND |
| *LacS* | 10.6±2.54 | 15.3±8.90 | 11.8±4.11 | 4.1±1.20^b^ | 0.16±0.06 | 0.5±0.18 | 0.6±2.30 | ND |
| *P-value* | *0.437* | *0.150* | *0.153* | *0.020* | *0.284* | *0.645* | *0.116* | - |

^1^Values (µmol/g) are presented as least squares means.

^a-b^Values within a column without a common superscript differ *P* < 0.05.

ND: Not detected

**Table S3.** Effects of probiotic administration on short chain fatty acid concentration in jejunal and caecal contents (Trial 2).

|  | Acetic acid | Succinic acid | Butyric acid | Isovaleric acid | Valeric acid | Lactic acid |
| --- | --- | --- | --- | --- | --- | --- |
| ***Day 7*** |  |  |  |  |  |  |
| ***Jejunum*** |  |  |  |  |  |  |
| Control | 0.1±0.06 | 0.3±0.09 | ND | ND | ND | 2.6±0.65 |
| Antibiotic | 0.1±0.07 | 0.3±0.11 | ND | ND | ND | 5.5±1.03 |
| Probiotic 0-10d | 0.1±0.07 | 0.2±0.11 | ND | ND | ND | 3.8±0.84 |
| Probiotic 0-35d | 0.3±0.07 | 0.3±0.11 | ND | ND | ND | 2.1±1.03 |
| *P-value* | *0.304* | *0.954* | - | - | - | *0.200* |
| ***Caecum*** |  |  |  |  |  |  |
| Control | 3.6±1.40 | 1.1±0.40 | 0.9±0.39 | ND | ND | ND |
| Antibiotic | 9.4±1.40 | 1.3±0.40 | 2.6±0.40 | ND | ND | ND |
| Probiotic 0-10d | 3.3±1.40 | 0.9±0.40 | 1.2±0.39 | ND | ND | ND |
| Probiotic 0-35d | 5.5±1.56 | 0.9±0.44 | 1.6±0.39 | ND | ND | ND |
| *P-value* | *0.082* | *0.843* | *0.134* | - | - | - |
| ***Day 21*** |  |  |  |  |  |  |
| ***Jejunum*** |  |  |  |  |  |  |
| Control | 0.2±0.06 | 0.2±0.07 | ND | ND | ND | 0.6±0.25^d^ |
| Antibiotic | 0.3±0.06 | 0.1±0.06 | ND | ND | ND | 5.4±0.25^a^ |
| Probiotic 0-10d | 0.2±0.06 | 0.2±0.07 | ND | ND | ND | 1.6±0.23^b^ |
| Probiotic 0-35d | 0.3±0.06 | 0.4±0.06 | ND | ND | ND | 2.5±0.24^c^ |
| *P-value* | *0.947* | *0.114* | - | - | - | *0.010* |
| ***Caecum*** |  |  |  |  |  |  |
| Control | 12.8±2.98^ab^ | 2.3±3.39 | 6.6±1.24 | 0.2±0.02 | 0.4±0.11 | ND |
| Antibiotic | 18.1±2.66^a^ | 3.3±3.39 | 3.6±1.10 | 0.1±0.03 | 0.5±0.12 | ND |
| Probiotic 0-10d | 4.2±2.66^b^ | 7.7±3.09 | 1.6±1.10 | 0.01±0.03 | 0.3±0.14 | ND |
| Probiotic 0-35d | 11.7±3.44^ab^ | 0.8±3.79 | 5.5±1.43 | 0.01±0.03 | 0.5±0.16 | ND |
| P-*value* | *0.034* | *0.514* | *0.073* | *0.250* | *0.834* | - |

^1^Values (µmol/g) are presented as least squares means.

^a-b^Values within a column without a common superscript differ *P* < 0.05.

ND: Not detected

1. [↑](#footnote-ref-2)
